# Electrically programmable probabilistic bit anti-correlator on a nanomagnetic platform

**DOI:** 10.1038/s41598-020-68996-y

**Published:** 2020-07-23

**Authors:** Mason T. McCray, Md Ahsanul Abeed, Supriyo Bandyopadhyay

**Affiliations:** Department of Electrical and Computer Engineering, Virginia Commonwealth University, Richmond, VA 23284 USA

**Keywords:** Engineering, Electrical and electronic engineering

## Abstract

Execution of probabilistic computing algorithms require electrically programmable stochasticity to encode arbitrary probability functions and controlled stochastic interaction or correlation between probabilistic (p-) bits. The latter is implemented with complex electronic components leaving a large footprint on a chip and dissipating excessive amount of energy. Here, we show an elegant implementation with just two dipole-coupled magneto-tunneling junctions (MTJ), with magnetostrictive soft layers, fabricated on a piezoelectric film. The resistance states of the two MTJs (high or low) encode the p-bit values (1 or 0) in the two streams. The first MTJ is driven to a resistance state with desired probability via a current or voltage that generates spin transfer torque, while the second MTJ’s resistance state is determined by dipole coupling with the first, thus correlating the second p-bit stream with the first. The effect of dipole coupling can be varied by generating local strain in the soft layer of the second MTJ with a local voltage (~ 0.2 V) and that varies the degree of anti-correlation between the resistance states of the two MTJs and hence between the two streams (from 0 to 100%). This paradigm generates the anti-correlation with “wireless” dipole coupling that consumes no footprint on a chip and dissipates no energy, and it controls the degree of anti-correlation with electrically generated strain that consumes minimal footprint and is extremely frugal in its use of energy. It can be extended to arbitrary number of bit streams. This realizes an “all-magnetic” platform for generating correlations or anti-correlations for probabilistic computing. It also implements a simple 2-node Bayesian network.

## Introduction

There is an emerging focus on “probabilistic computing” with probabilistic (*p-*) *bits* encoded in the resistance states of magneto-tunneling junctions (MTJs) whose soft layers are nanomagnets possessing low energy barriers^1^. This paradigm has been shown to be successful in solving computationally-hard problems such as factorization^2^, combinatorial optimization^3^, population coding^4^, invertible logic^5^, and has been used in restricted Boltzmann machines^6^ and belief networks^7^. p-bit based computing requires stochastic interaction and controlled correlation between p-bits. Controlled correlations are also needed for some graphical circuit models of stochastic computing, computer vision and image processing applications^8–12^. These correlations are typically generated with the aid of complex electronic hardware that may involve shift registers, Boolean logic gates, random number generators, multiplexers, binary counters, comparators, etc. or microcontrollers for generating synaptic weights^2–5^. They consume large areas on a chip, dissipate enormous amounts of energy, and are expensive to implement. In this paper, we show how to implement an ultra-compact and ultra-energy-efficient p-bit correlator with dipole-coupled MTJs fabricated on a piezoelectric film, with each MTJ hosting a p-bit. The dipole coupling correlates the two p-bits and the degree of correlation can be varied from no correlation to perfect anti-correlation by straining one of the MTJs with a small voltage (~ 0.2 V) which modulates the effect of dipole coupling. *This results in an “all-magnetic” platform for generating controlled stochastic interaction in probabilistic computing that does not require any active electronic component*. Additionally, dipole coupling is a “wireless” interaction and hence does not have any ohmic dissipation, while the energy expended to generate the strain is very low (~ 250 aJ). These features make the correlator extremely energy-efficient compared to other (electronic) renditions.

## Operating principle

**Figure 1:**
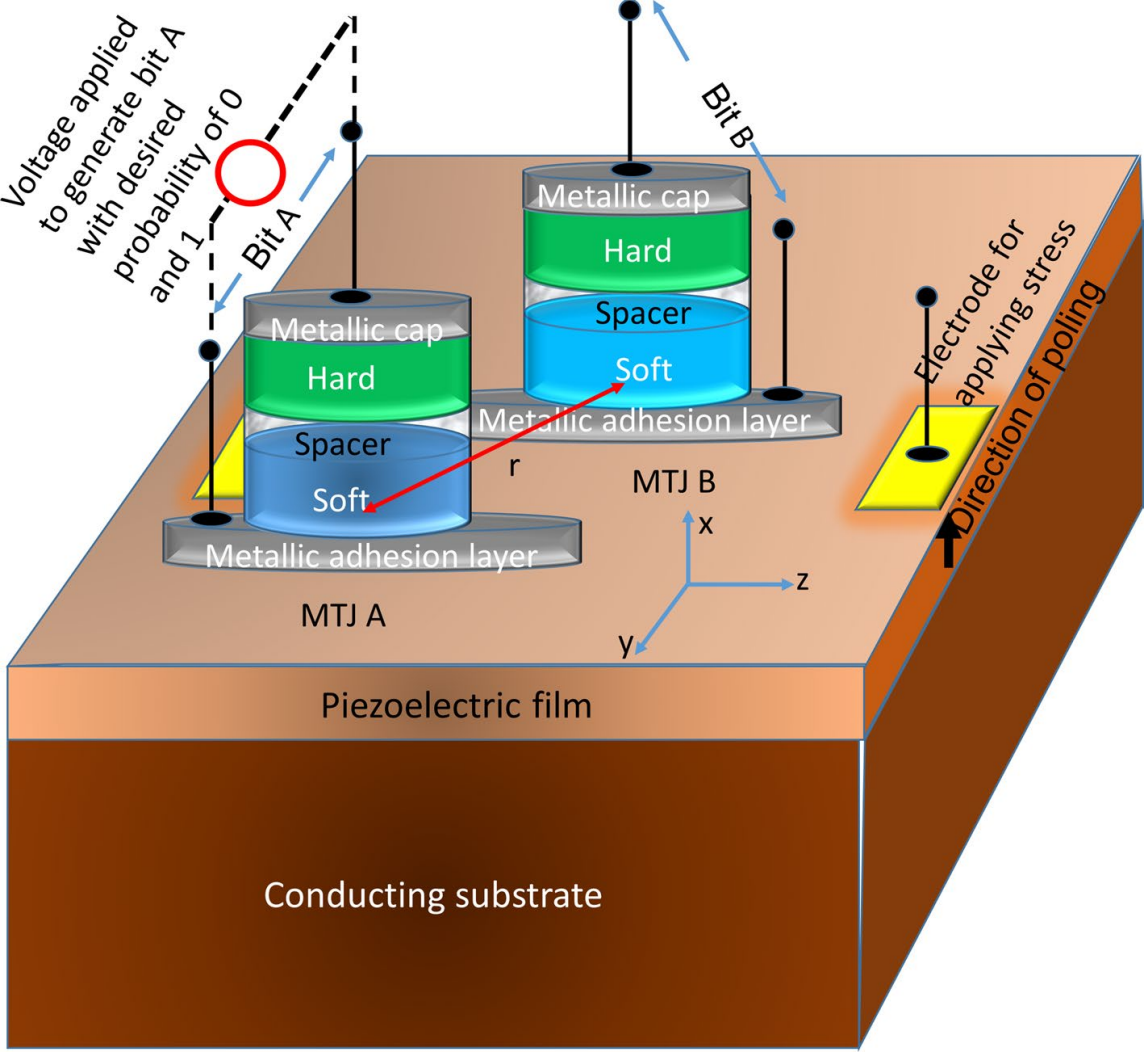
A magneto-elastic system for generating two random bits with controlled amount of correlation. The metallic adhesion layer is thin enough to not impede strain transfer from the piezoelectric film to the soft layers.

Figure 1 shows the system being discussed. It consists of two magneto-tunneling junctions (MTJs A and B) of elliptical cross-sections whose major and minor axes are mutually parallel. Both the hard and soft layers of the MTJs have in-plane magnetic anisotropy, but the principle described here would not be affected if they had perpendicular magnetic anisotropy. Both soft layers are amorphous, except perhaps at the interfaces with the spacer layers, and hence possess virtually no magneto-crystalline anisotropy. The MTJs are fabricated on a poled piezoelectric film deposited on a conducting substrate and are placed close enough to each other that their soft layers experience significant dipole coupling. Two electrodes are delineated on the piezoelectric film (flanking the soft layer of MTJ B *only*) with appropriate dimensions and spacing to generate local biaxial strain (tensile along the major axis and compressive along the minor axis, or vice versa) underneath the magnetostrictive soft layer of MTJ B (overcoming any substrate clamping) when a voltage of appropriate polarity is applied between the electrodes and the bottom conducting substrate^13,14^. By varying this voltage, we can vary the magnitude of the strain experienced by the magnetostrictive soft layer of MTJ B. The soft layer of MTJ A will also experience some strain in this process, but its magnetization will be determined primarily by the voltage applied across it, which drives a spin-polarized current through it that generates spin transfer torque (STT). The STT will override any strain effect because the latter is much weaker. It will also override any effect of dipole coupling from MTJ B.

No voltage is applied across MTJ B to produce any STT in MTJ B. The state of MTJ B is determined solely by the effect of dipole coupling exerted on it by MTJ A and is hence correlated or anti-correlated with the state of MTJ A to varying degrees (the “degree” being varied by varying the effect of dipole coupling with local strain applied selectively to MTJ B).

The purpose of the strain is to modulate the energy barrier in a soft layer. The strain anisotropy energy for uniaxial strain *σ* applied along the easy axis (major axis of the ellipse) is $\left( {{3 \mathord{\left/ {\vphantom {3 2}} \right. \kern-\nulldelimiterspace} 2}} \right)\lambda_{s} \sigma \Omega$, where $\lambda_{s}$ is the saturation magnetostriction and Ω is the volume of the soft layer. As long as the product $\lambda_{s} \sigma$ is negative, strain will depress the energy barrier. Thus, for a material with positive magnetostriction (e.g. Terfenol-D, Galfenol), uniaxial compressive strain along the major axis (or uniaxial tensile strain along the minor axis) will depress the energy barrier, whereas uniaxial tensile strain along the major axis (or uniaxial compressive strain along the minor axis) will raise the energy barrier.

The sole purpose of the conducting substrate in Fig. 1 is to reduce the voltage dropped over the substrate and hence reduce the voltage that needs to be applied at the surface electrodes to induce a given strain in the soft layer of MTJ B. The conducting substrate can be n^+^-silicon^15^, which will make this device CMOS-compatible.

The values of the two p-bits (*A* and *B*) are encoded in the resistance states of the two MTJs—high resistance is bit 1 and low resistance bit 0. These resistance states can be converted to corresponding voltage or current states with a constant current source or a constant voltage source, respectively, for reading the bit.

To generate the p-bit *A* with a desired probability of 0 or 1, we apply a voltage across MTJ A with appropriate magnitude and polarity, which drives a spin polarized current through its soft layer, applies a spin transfer torque^16,17^ and takes MTJ A to the low or the high resistance state with the desired probability. This allows us to generate the probability *P*(*x*) [*x* = 0, 1]. This modality works regardless of whether the MTJ has in-plane magnetic anisotropy (see supplementary material) or perpendicular magnetic anisotropy^14^ (see Fig. 2 of ref.^14^). For example, if we choose to make the-p-bit *A* = 0 with 70% probability (and hence *A* = 1 with 30% probability), we will first initialize the resistance state of MTJ A to “low” (corresponding to *A* = 0) with a very large STT current with appropriate direction of spin polarization (which will make the probability of MTJ A going to the low resistance state very close to 100%) and then inject another STT current of appropriate spin polarization into MTJ A to make its resistance state flip with 30% probability, resulting in the desired probability of *P(A* = 0) = 1 − *P*(*A* = 1) = 0.7. In the Supplementary Material, we show the calculated *P*(*A* = *1*) probability as a function of the spin polarized current after the magnetization had been initialized to the state *A* = 0 with a large spin polarized current.

**Figure 2:**
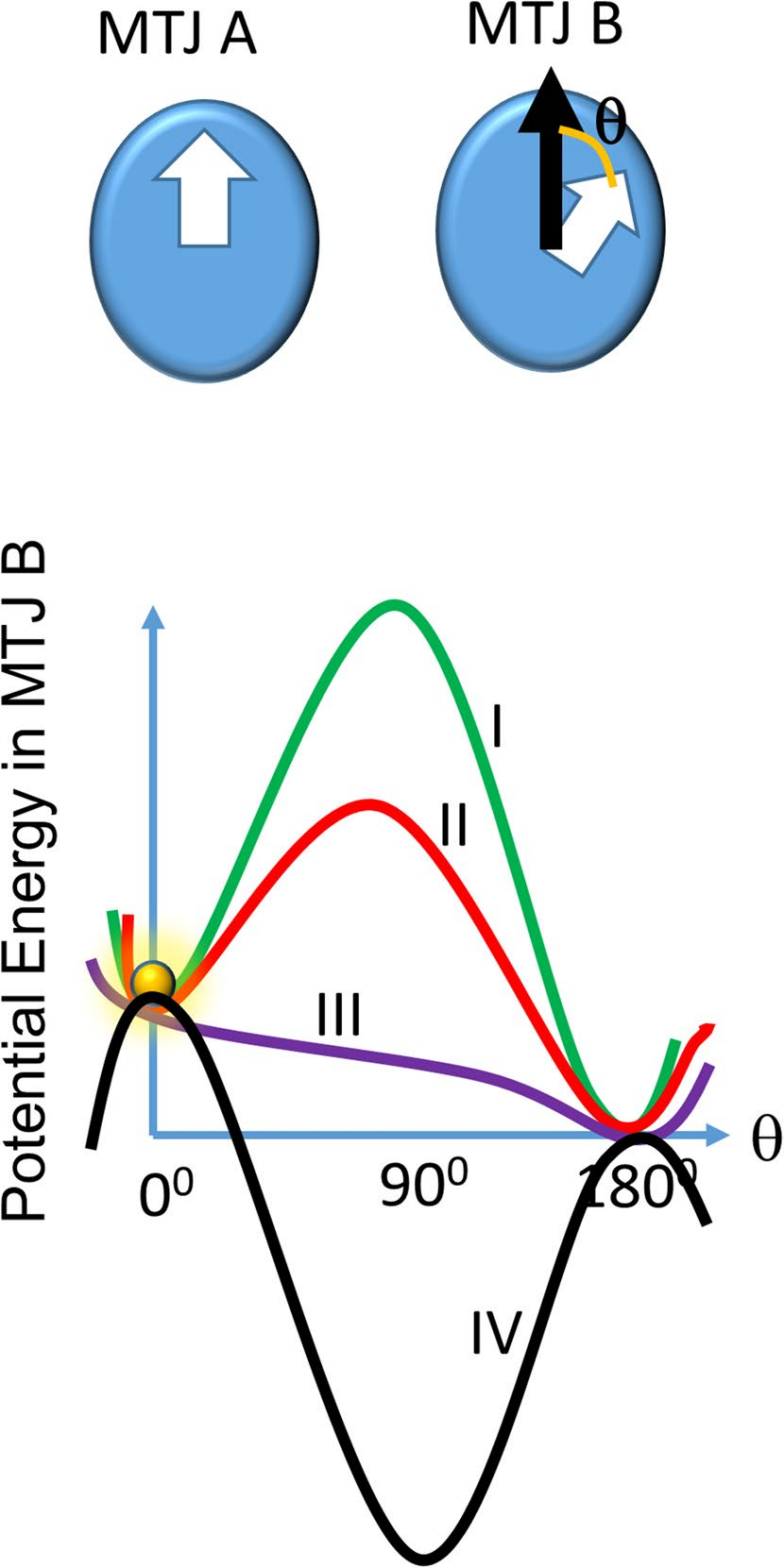
Potential energy as a function of magnetization orientation θ within the soft layer of MTJ B in the presence of dipole coupling with MTJ A whose magnetization is oriented as shown in the top panel. The potential profile is asymmetric because of the dipole coupling. (I) No stress applied, (II) sub-critical stress applied, (III) critical stress applied, and (IV) super-critical stress applied. The ball indicates the initial magnetization state of the soft layer of MTJ B.

To *anti*-*correlate* the p-bit *B* with p-bit *A controllably*, we do not inject any current into MTJ B. Instead, we rely on the dipole coupling between the soft layers of MTJ A and MTJ B to set the resistance state of MTJ B, and hence the value of the Bit B, depending on the value of the p-bit A. This generates *engineered* conditional probability *P*(*B|A*) or *controlled* stochastic coupling/correlation between p-bits *A* and *B*. One would expect that dipole coupling between the MTJs would always make the magnetization of the soft layer of MTJ B become antiparallel to the magnetization of the soft layer of MTJ A, resulting in perfect anti-correlation between p-bits *A* and *B*, but this does not happen unless the dipole coupling is extremely strong. There is a shape anisotropy energy barrier within the soft layer of MTJ B, which will *have to be overcome* by dipole coupling to make its magnetization rotate from its initial orientation (if it was not initially antiparallel) to become antiparallel to that of the soft layer of MTJ A. If this energy barrier is too large and cannot be overcome by dipole coupling, then p-bit *B* will be completely uncorrelated with p-bit *A* (0% anti-correlation) since it will not be responsive to the state of bit *A*. On the other hand, if the energy barrier is so small that dipole coupling can always overcome it, then p-bit *B* will always be perfectly anti-correlated with p-bit *A* (100% anti-correlation). For intermediate barrier heights, the anti-correlation will be between 0 and 100%. By *varying the barrier height in the soft layer of MTJ B with local strain*, we can vary the anti-correlation between 0 and 100%. We elucidate this in Fig. 2.

The MTJs that we consider have elliptical soft layers made of Terfenol-D. The rationale for choosing this material is that it has very high magnetostriction and hence the soft layer would experience a relatively large strain anisotropy energy even when the strain (or stress) generated in it is small. This will reduce the amount of voltage required to be applied at the top electrodes in Fig. 1 to modulate the energy barrier in the soft layer of MTJ B (as shown in Fig. 2). The shape anisotropy energy barrier in the soft layer of MTJ B (and hence the energy barrier in the absence of strain) is 378 kT at room temperature. The dipole coupling energy can be estimated from the relation $E_{{{\text{dipole}}}} = \frac{{\mu_{0} M_{s}^{2} \Omega^{2} }}{{4\pi r^{3} }}$ where µ_o_ is the permeability of free space, *M*_*s*_ is the saturation magnetization of the soft layer, Ω is its volume and *r* is the center-to-center separation between the soft layers of the two MTJs. For our case, this quantity would be 82 kT for a separation of 150 nm, which is much less than the energy barrier of 378 kT in the soft layer of MTJ B. Therefore, dipole coupling, by itself, cannot overcome the energy barrier. That means if no strain is applied to depress the energy barrier of 378 kT, the magnetization in the soft layer of MTJ B will be independent of that in MTJ A and there will be 0% anti-correlation between bits A and B. By applying varying stress, we can depress the energy barrier by different amounts and thus generate various degrees of anti-correlation between bits *A* and *B*.

To elucidate the principle further, in the top panel of Fig. 2, we show the top views of the soft layers of the two MTJs. Suppose the magnetization of the soft layer of MTJ B was initially at θ = 0° (see the top panel of Fig. 2 for the definition of θ) and the magnetization of MTJ A became oriented along θ = 0° by the voltage *V*_*A*_ applied across it to generate STT. This places the two soft layers in the parallel configuration. The potential energy profile in the soft layer of MTJ B as a function of its magnetization orientation will look like curve I in the lower panel in Fig. 2 (it is asymmetric because of the dipole coupling). The MTJ B will be stuck in the in the metastable state (local energy minimum) at θ = 0° (keeping MTJ A and MTJ B parallel) and not be able to transition to the global energy minimum at θ = 180° (where MTJ A and MTJ B will be mutually anti-parallel) because of the intervening potential energy barrier. In this case, p-bit *B* will not be able to respond to p-bit *A* and the two p-bits will be *uncorrelated*. However, if we apply varying degree of stress to the soft layer of MTJ B, then we will depress the energy barrier by varying degrees and thus be able to tune the probability that the system can transition to the ground state at θ = 180° where the two MTJs will be anti-parallel and hence anti-correlated. Thus, by varying the voltage applied to the two electrodes in Fig. 1, which varies the stress produced in the soft layer of MTJ B, we can *vary the anti-correlation between p- bits A and B* from no (0%) anti-correlation to perfect (100%) anti-correlation.

## Maximum anti-correlation at critical stress

In Fig. 2, we see that different amounts of stress affect the energy barrier in different ways. We define “critical stress” as the amount of stress that *just* erodes the energy barrier completely, but does not invert it to create a potential well at θ  = 90° (Case III in Fig. 2). Sub-critical stress (Cases I and II) does not erode the potential barrier completely and super-critical stress (Case IV) inverts the potential barrier. Neither sub-critical, nor super-critical stress will make the probability of transitioning from θ = 0° to θ = 180° maximum. If the barrier is inverted by (super-critical) stress, then the magnetization will locate to θ  = 90° as long as the stress is on. After stress removal, the magnetization will find itself at an energy maximum since the potential profile will revert to Case I, and thereafter, the magnetization will have some probability of transitioning to θ = 180° (flipping), but also a substantial probability of transitioning back to θ = 0°, i.e. not flipping. Therefore, super-critical stress does not guarantee a very high probability of flipping the magnetization of MTJ B in response to the dipole coupling effect of MTJ. Looking at the potential profiles in Fig. 2, one would understand that the maximum likelihood that the magnetization of MTJ B will flip in response to the magnetization state of MTJ A (i.e. the ball shown in Fig. 2 will roll down smoothly from θ = 0° to θ = 180°) will occur when the applied stress is the *critical stress*, which just erodes the potential barrier, but does not invert it. Thus, *critical stress will result in the maximum anti-correlation* between bits A and B.

## Simulation results

We have simulated the magneto-dynamics within the soft layers of both MTJ A and MTJ B in the presence of thermal noise, dipole coupling, stress applied to MTJ B and voltage applied to MTJ A by solving *coupled* stochastic Landau-Lifshitz-Gilbert (s-LLG) equations for the two soft layers.

In the case of MTJ A, the p-bit state *A* is determined primarily by the spin polarized current that flows through the MTJ when a voltage *V*_*A*_ is applied across it. This voltage generates spin transfer torque (STT). We have, of course, also considered the effect of dipole coupling with MTJ B. In the case of MTJ B, there is no voltage applied across it (hence no STT), and its p-bit state is determined by dipole coupling with MTJ A *and* stress which modulates the energy barrier within it.

The s-LLG equation is

1 $$\begin{aligned} \frac{{d\vec{m}\left( t \right)}}{dt} & = - \gamma \vec{m}\left( t \right) \times \vec{H}_{{{\text{total}}}} \left( t \right) + \alpha \left( {\vec{m}\left( t \right) \times \frac{{d\vec{m}\left( t \right)}}{dt}} \right) \\ & \quad + a\vec{m}\left( t \right) \times \left( {\frac{{\eta \vec{I}_{s} \left( t \right)\mu_{B} }}{{qM_{s} \Omega }} \times \vec{m}\left( t \right) \, } \right) + b\frac{{\eta \vec{I}_{s} \left( t \right)\mu_{B} }}{{qM_{s} \Omega }} \times \vec{m}\left( t \right) \\ & {\text{where}} \\ \hat{m}\left( t \right) & = m_{x} \left( t \right)\hat{x} + m_{y} \left( t \right)\hat{y} + m_{z} \left( t \right)\hat{z} \, \quad \, \left[ {m_{x}^{2} \left( t \right) + m_{y}^{2} \left( t \right) + m_{z}^{2} \left( t \right) = 1} \right] \\ \vec{H}_{{{\text{total}}}} & = \vec{H}_{{{\text{demag}}}} + \vec{H}_{{{\text{stress}}}} + \vec{H}_{{{\text{dipole}}}} + \vec{H}_{{{\text{thermal}}}} \\ \vec{H}_{{{\text{demag}}}} & = - M_{s} N_{d - xx} m_{x} \left( t \right)\hat{x} - M_{s} N_{d - yy} m_{y} \left( t \right)\hat{y} - M_{s} N_{d - zz} m_{z} \left( t \right)\hat{z} \, \\ \vec{H}_{{{\text{stress}}}} & = \frac{3}{{\mu_{0} M_{s} }}\left( {\lambda_{s} \sigma_{zz} \left( t \right)m_{z} \left( t \right)} \right)\hat{z} \\ \vec{H}_{{{\text{dipole}}}} & = - \frac{{M_{s} \Omega }}{{4\pi r^{3} }}\left[ {\tilde{m}_{x} \hat{x} - 2\tilde{m}_{y} \hat{y} + \tilde{m}_{z} \hat{z}} \right] \, \left[ \begin{gathered} {\text{tilda represents magnetization }} \hfill \\ {\text{of the neighboring nanomagnet}} \hfill \\ \end{gathered} \right] \\ \vec{H}_{{{\text{thermal}}}} & = \sqrt {\frac{2\alpha kT}{{\gamma \left( {1 + \alpha^{2} } \right)\mu_{0} M_{s} \Omega \left( {\Delta t} \right)}}} \left[ {G_{{_{{\left( {0,1} \right)}} }}^{x} \left( t \right)\hat{x} + G_{{_{{\left( {0,1} \right)}} }}^{y} \left( t \right)\hat{y} + G_{{_{{\left( {0,1} \right)}} }}^{z} \left( t \right)\hat{z}} \right] \\ \end{aligned}$$

The last term in the right hand side of Eq. (1) is the field-like spin transfer torque and the second to last term is the Slonczewski torque. The coefficients *a* and *b* depend on device configurations and following^13^, we will use the values $a = 1, \, b = 0.3$. Here $\hat{m}\left( t \right)$ is the time-varying magnetization vector in the nanomagnet normalized to unity, *m*_*x*_*(t)*, *m*_*y*_*(t)* and *m*_*z*_*(t)* are its time-varying components along the x-, y- and z-axis, $\vec{H}_{{{\text{demag}}}}$ is the demagnetizing field in the soft layer due to shape anisotropy and $\vec{H}_{{{\text{thermal}}}}$ is the random magnetic field due to thermal noise. The different parameters in Eq. (1) are: $\gamma = {{2\mu_{B} \mu_{0} } \mathord{\left/ {\vphantom {{2\mu_{B} \mu_{0} } \hbar }} \right. \kern-\nulldelimiterspace} \hbar }$ (gyromagnetic ratio), *α* is the Gilbert damping constant, $\mu_{0}$ is the magnetic permeability of free space, *M*_*s*_ is the saturation magnetization of the magnetostrictive soft layer, *kT* is the thermal energy, Ω is the volume of the nanomagnet given by $\Omega = \left( {{\pi \mathord{\left/ {\vphantom {\pi 4}} \right. \kern-\nulldelimiterspace} 4}} \right)a_{1} a_{2} a_{3} \,$, *a*_1_ = major axis, *a*_2_ = minor axis, and *a*_3_ = thickness, Δ*t* is the time step used in the simulation (0.1 ps), and $G_{{_{{\left( {0,1} \right)}} }}^{x} \left( t \right)$, $G_{{_{{\left( {0,1} \right)}} }}^{x} \left( t \right)$ and $G_{{_{{\left( {0,1} \right)}} }}^{x} \left( t \right)$ are three uncorrelated Gaussians with zero mean and unit standard deviation^18^. The quantities $N_{d - xx} ,N_{d - yy} ,N_{d - zz} \, \left[ {N_{d - xx} + N_{d - yy} + N_{d - zz} = 1} \right]$ are calculated from the dimensions of the nanomagnet following the prescription of ref.^19^. We assume that the charge current injected into the nanomagnet is $\vec{I}_{s} \left( t \right)$ and that the spin polarization in the current is *η*. The spin current is given by $\eta \vec{I}_{s} \left( t \right) = \eta \left| {\vec{I}_{s} \left( t \right)} \right|\hat{z}$ where $\hat{z}$ is the unit vector along the major axis as shown in Fig. 1. The various parameters for the simulation are given in Table 1.

**Table 1:** Parameters used in the simulation.

Parameters	Values
Saturation magnetization (*M*_*s*_)	8 × 10^5^ A/m
Gilbert damping (α)	0.1
Temperature (T)	300 K
Spin polarization (*η*)	0.3
Major axis (a1)	100 nm
Minor axis (a2)	90 nm
Thickness (a3)	15 nm

Since it is difficult to incorporate biaxial strain in the s-LLG equation, we approximate it as a uniaxial stress directed along the major axis of the elliptical soft layer (z-axis; see Fig. 1). The uniaxial stress is written as *σ*_*zz*_ and *λ*_*s*_ is the saturation magnetostriction of Terfenol-D, which is 600 ppm^20^.

The voltage applied across MTJ A injects/extracts spin polarized carriers into its soft layer with their spins polarized in the $\pm z$ direction and *η* is the spin polarization of the current, which we choose as 30%. We define a *correlation parameter* as

2 $$C = \left\langle {A \times B} \right\rangle$$

where *A* is the value of p-bit A (either + 1 or − 1) and *B* is the value of p-bit B (also either + 1 or − 1). We use − 1 instead of 0 to represent the logic complement of 1. The angular brackets denote ensemble average. The ensemble averaging is carried out over 1,000 switching trajectories generated in our simulator. The ensemble average *C* (correlation parameter) is a *measure* of the degree of anti-correlation between p-bits *A* and *B*. The value *C* = − 1 indicates perfect (100%) anti-correlation and *C* =  + 1 indicates no (0%) anti-correlation, while C = 0 indicates 50% anti-correlation. Any intermediate value indicates partial anti-correlation.

The switching trajectories are generated in the following manner. For MTJ A, we assume that the magnetization of its soft layer is initially pointing in the + z-direction. We then apply a voltage of appropriate polarity that results in passing a spin polarized current, with spin polarization in the –z-direction and magnitude varying between 30 and 40 mA (above the critical current for switching) and simulate the magneto-dynamics of the soft layer of MTJ A which yields its magnetization as a function of time $m_{A} \left( t \right)$ At the same time, we simulate the magneto-dynamics in the soft layer of MTJ B (which is initially magnetized in the -z-direction) to find its magnetization $m_{B} \left( t \right)$ as a function of time. The magneto-dynamics of the two soft layers are coupled through the dipole coupling term;$m_{A} \left( t \right)$ determines $m_{B} \left( t \right)$ and vice versa. The simulations are carried out until the magnetizations of both soft layers reach steady state, at which point they are either approximately parallel or antiparallel. If they are parallel, in which case dipole coupling was not effective and *B* remained oblivious of the state of *A*, then $A \times B$ =  + 1, whereas if they are antiparallel, in which case dipole coupling was effective to correlate *B* with *A*, then $A \times B$ = − 1. We average the value of $A \times B$ over the 1,000 switching trajectories to calculate the correlation parameter *C* defined in Eq. (2).

**Figure 3:**
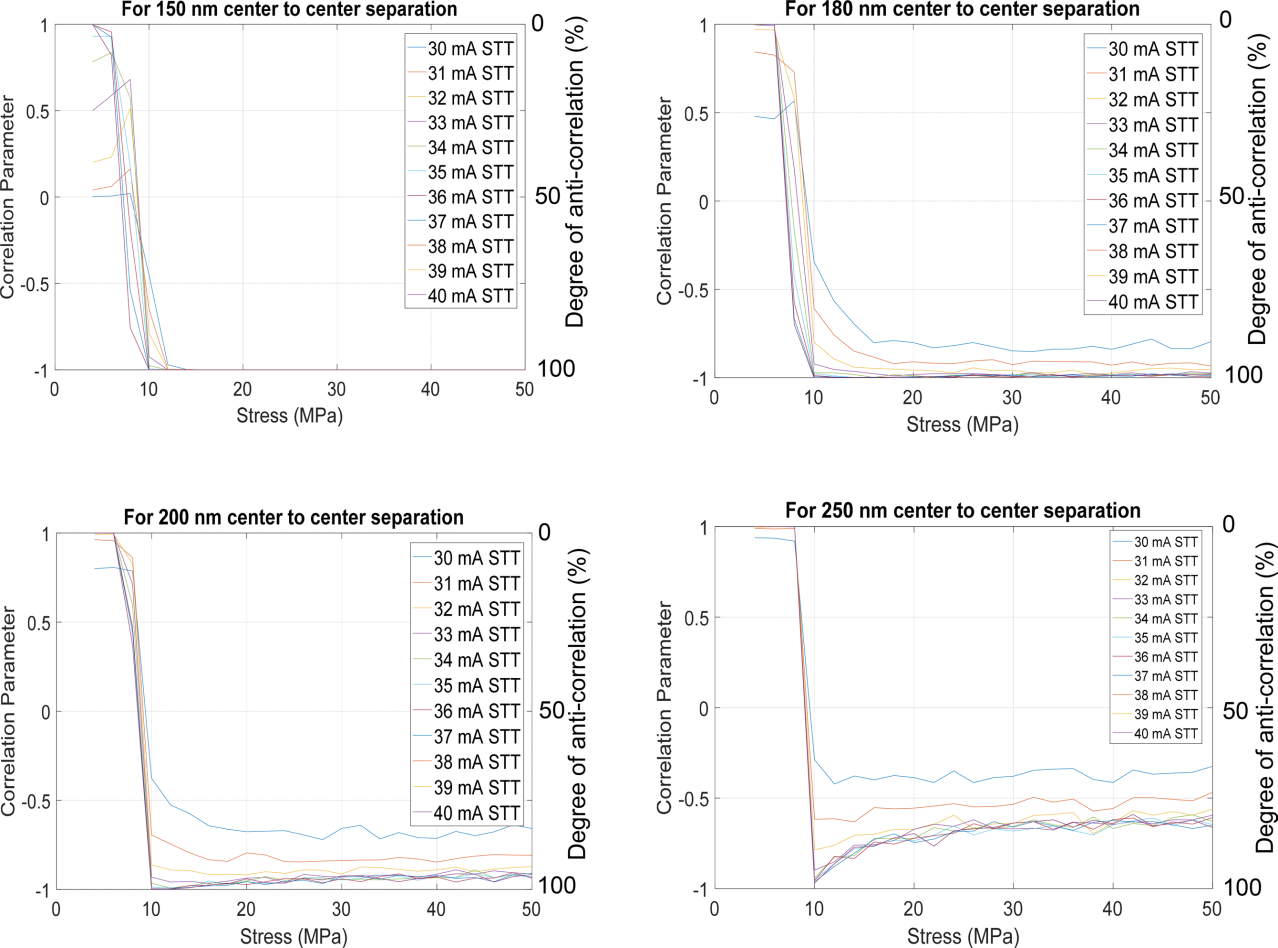
Correlation parameter *C* and the degree of anti-correlation between bits *A* and *B* as a function of stress for different spin polarized currents and for four different spatial separations between MTJ A and MTJ B.

In Fig. 3, we show plots of the correlation parameter as a function of the stress applied to the soft layer of MTJ B for different amplitudes of the current pulse injected into MJTJ A (no current is injected into MTJ B). The results are shown for four different center-to-center separations between the soft layers of MTJ A and MTJ B (and hence four different strengths of the dipole coupling; the dipole coupling strength varies as the inverse third power of the separation). There are several features of interest found in this figure. First and foremost, we find that we can vary the correlation parameter *C* from + 1 (no anti-correlation or 0% anti-correlation) to − 1 (perfect anti-correlation or 100% anti-correlation) continuously by varying the stress applied locally to the soft layer of MTJ B with the voltage impressed between the shorted electrodes in Fig. 1 and the conducting substrate. Thus, we can control the anti-correlation between p-bits *A* and *B* to be anything between 0 and 100% with an external voltage. We can do this for any pair of bits in two bit streams and it is obvious that we can extend it to more than two bit streams by having additional dipole coupled MTJs. We can also have different spacings between the MTJs to achieve different anti-correlations between different bit streams. This paradigm allows perfect tunability of the degree of anti-correlation.

There are other interesting features in Fig. 3. The anti-correlation decreases with increasing separation between the MTJs (i.e. decreasing dipole coupling strength), which is expected since the anti-correlation is caused by dipole coupling. The correlation parameter *C* also depends on the current injected into MTJ A because this current determines the probability with which the state of p-bit A is set. A low current may not succeed in “writing” the state of p-bit A with high probability and a manifestation of that is the failure to reach perfect anti-correlation, even at high stress values, when the current is low. This feature becomes increasingly prominent with increasing separation between the MTJs (decreasing dipole coupling strength). The current densities used here are high (~ 5 $\times$ 10^12^ A/m^2^), but this is a consequence of the materials and geometry chosen. A lower energy barrier in the soft layers will reduce the current density. The current density can also be lowered by an order of magnitude if we initialize the magnetizations of both soft layers to point along the minor axis of the ellipses (along the hard axes) with a global in-plane magnetic field. This will reduce the current density by more than an order of magnitude as shown in Fig. S2(b) in the Supplementary Material. The downside of this approach is the requirement of the in-plane magnetic field. It will take some energy dissipation to generate this field on-chip, but it is a global magnetic field and when the energy cost is amortized over numerous MTJs, the cost per bit would be negligible. Other approaches to reducing the current density, such as by aligning the magnetizations of all soft layers along the hard axis (minor axis) with a global stress field, may be possible, but they are not an objective of this work.

Let us assume that we are able to reduce the current density by an order of magnitude to about 5 $\times$ 10^11^ A/m^2^ by adopting the above approach. Low resistance area product of 10 Ω-µm^2^ has been demonstrated in MTJs^21^ and the present trend to reduce it further by using low bandgap spacer layers like ScN (in lieu of the traditional MgO spacer layer) may reduce it further by an order of magnitude. Therefore, the power dissipated to write a bit into an MTJ with the above approach will be ~ 450 µW. If the write pulse has a duration of 1 ns^21^, then the energy dissipated to write a bit will be ~ 450 fJ. We can reduce this further, to perhaps 100 fJ, by reducing the energy barrier in the soft layer from 378 kT to a smaller value by reducing the eccentricity of the ellipse (smaller shape anisotropy energy barrier).

**Figure 4:**
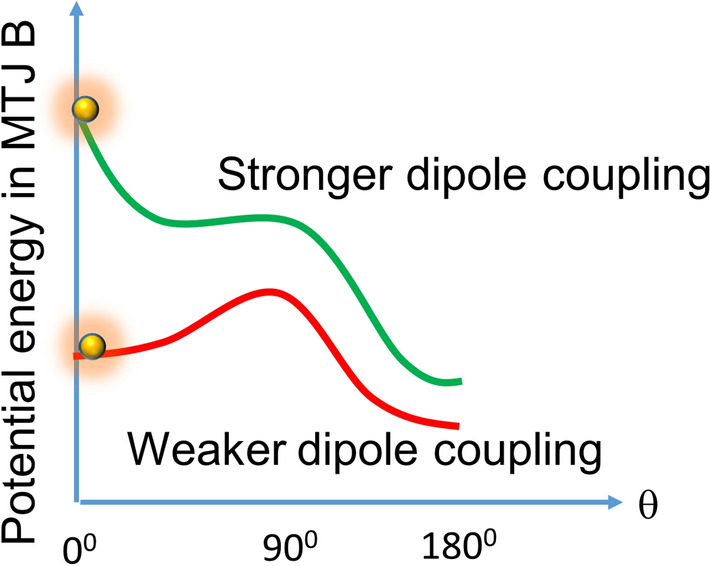
Potential energy profile (energy as a function of magnetization orientation in the soft layer of MTJ B) for strong and weak dipole coupling.

Another very interesting feature clearly observed at higher inter-MTJ separations (bottom panels in Fig. 3) is that the anti-correlation becomes maximum at a certain stress value (note the non-monotonic behavior). This is the “critical stress” discussed in Section III and we see it clearly in these cases. In the case of 250 nm center-to-center separation, the critical stress is about 10 MPa. Why the critical stress feature is more prominent at larger inter-MTJ separation (weaker dipole coupling) is also easy to understand by looking at Fig. 4. When the separation is larger (dipole coupling weaker), the energy difference between the local and global minima in the potential profile of the soft layer of MTJ B is smaller. In that case, it will be more important to completely erode the potential barrier without inverting it, in order to make the soft layer switch (ball to roll down from local to global minimum) with very high probability. On the other hand, if the inter-MTJ separation is smaller (dipole coupling stronger), then the energy difference between the local and global minima will be larger and eroding the barrier completely will not be so critical to ensure that the soft layer switches to reach the global minimum.

Finally, we can roughly estimate how much voltage will be required to generate the stress of 10 MPa in the soft layer of MTJ B needed to vary the correlation from no correlation to nearly perfect anti-correlation (see Fig. 3). The electric field *E* needed to produce a uniaxial strain *ε*_*zz*_ in the piezoelectric film is given by

3 $$E = \frac{{\varepsilon_{zz} }}{{d_{31} }}$$

where *d*_*31*_ is the piezoelectric coefficient of the piezoelectric film. We will assume that the strain generated in the piezoelectric is fully transferred to the soft layer, which is a reasonable assumption as long as the thickness of the soft layer is much less than that of the piezoelectric film. If we assume that the piezoelectric film is made of Pb(Mg_1/3_Nb_2/3_)O_3_-PbTiO_3_ (PMN-PT), and the soft layer is made of Terfenol-D, then using the values available in the literature for PMN-PT (*d*_*31*_ = 1,200 pm/V^22^) and Terfenol-D (Young’s modulus *Y* = 45 GPa^23^), the electric field needed to produce a stress of 10 MPa in the soft layer of MTJ B is 0.18 MV/m. This can be produced by a voltage of 0.18 V dropped over a piezoelectric film of thickness 1 µm. Thus, the modulating voltage needs to be no more than ~ 0.2 V.

The energy dissipated in changing the anti-correlation from 0% (no correlation) to 100% (perfect anti-correlation) is at most *CV*^*2*^, where *C* is the capacitance of the electrode pairs and *V* is the voltage needed (0.18 V) to vary the correlation over the full range (0–100%). The relative dielectric constant of a piezoelectric material like PMN-PT depends on the crystallographic orientation of the film, but is typically ~ 1,000 ^24^. Assuming that the contact electrode areas are 1 µm $\times$ 1 µm and the piezoelectric film thickness is 1 µm, the capacitance *C* ~ 8 fF. Therefore, the energy dissipated in tuning the anti-correlation over its full range (0–100%) is a mere 260 aJ. That makes this an energy-efficient paradigm for tuning the anti-correlation among p-bits.

Let us now compare the total system energy cost per bit for the present scheme with a scheme that requires the use of microcontrollers and digital-to-analog converters to produce stochastic interaction among bits, as in ref.^8^. An Arduino microcontroller with no power LED dissipates about 116 µW at 16 MHz clock (period 62.5 ns)^25^. Therefore, the energy dissipation per bit in the microcontroller itself is about 7.25 pJ per bit, without accounting for other elements such as digital-to-analog converters. Thus, the system energy cost per bit in the present scheme (writing + anti-correlator) is about 100 fJ + 260 aJ = 100.26 fJ. In contrast, the energy cost per bit when using electronic elements like microcontrollers is more than 100 fJ + 7.25 pJ = 7.35 pJ. Therefore, the present scheme is orders of magnitude more energy efficient because of the extreme low power anti-correlator that replaces the energy-inefficient microcontrollers.

## Discussion

In this work, we have shown how to vary the anti-correlation between two p-bit streams *controllably*, resulting in a *tunable probability correlator*. The anti-correlation can be varied between 0 and 100% by strain-engineering the switching probability of the soft layer of the second MTJ under the influence of dipole coupling with a voltage of only ~ 0.2 V, resulting in an energy dissipation of ~ 260 aJ. Here, we have varied the “anti-correlation”, not the “correlation”. Dipole coupling, with the placement of MTJs considered here, would tend to align the magnetizations of the soft layers of MTJs A and B in opposite directions and hence by controlling dipole coupling with stress, we can only control the probability of the two magnetizations being anti-parallel, or the degree to which the two bits are anti-correlated. However, this is also equivalent to varying the correlation, since an anti-correlated bit stream can be converted to a correlated bit stream with the simple process of inversion.

It is also possible to have stress act as a “de-correlator”. One target application in stochastic computing is to generate two random bit streams with the same mean but with no correlation between the streams^28–30^. Suppose that we have two identical MTJs A1 and A2, each driven by independent random STT pulses with the same mean (so that they generate independent random bit streams with the same mean) but with some dipole coupling between them which generates some correlation between the bit streams A1 and A2. For many stochastic computing applications, this correlation, however slight, is undesirable and must be eliminated. We can de-correlate the bit streams by weakening or completely negating the dipole coupling effect by raising the energy barriers in the soft layers of both MTJs to the extent that the dipole coupling energy can never overcome the energy barriers to correlate the bits. Whether strain raises or lowers the energy barrier depends on the sign of the product $\lambda_{s} \sigma$ where $\lambda_{s}$ is the saturation magnetization and $\sigma$ is the uniaxial strain applied along the easy axis (major axis) of the elliptical soft layer. If the product is negative, then the barrier is lowered (anti-correlation increased) and if the product is positive, the barrier is raised (any correlation or anti-correlation reduced). For Terfenol-D, $\lambda_{s}$ is positive. Hence, uniaxial compressive strain applied along the major axis (or uniaxial tensile strain along the minor axis) will lower the barrier and increase anti-correlation. Reversing the signs of the stresses will raise the barrier and increasingly destroy any correlation or anti-correlation. Thus, by raising the barrier with electrically generated stress, we can destroy any correlation between the bits encoded in the two MTJs and thus stress can also act as a de-correlator. The degree of de-correlation is obviously electrically programmable since the stress is generated with an electrical voltage and its magnitude is controlled with the voltage.

One question that may arise in the case of the anti-correlator (or correlator) described here, implemented with MTJs A and B, is what happens when the dipole coupling effect in MTJ B is weak and so MTJ B does not respond to the state of MTJ A. Recall that only MTJ A is driven by a current or voltage pulse generating STT, while MTJ B has no independent electrical excitation and its state is determined solely by the dipole coupling effect exerted on it by MTJ A. In this case, MTJ B will be stuck in some state (either 0 or 1) and not produce any random bit stream. It must be understood that the purpose of MTJ B is solely to provide a correlated/anti-correlated bit stream. It is not an independent source of a random bit stream. If there is no correlation, then MTJ B serves no purpose and in that case it will not be used. If the purpose is to produce two uncorrelated bit streams, then we will use two MTJs A1 and A2, each driven by an independent random STT pulse, as described above.

Last, but not least, this construct effectively acts as a simple two-node Bayesian network for probability assessment and inference. Each MTJ acts as a network node that has a probability of being in one of two possible states (high and low resistance). MTJ A acts as the “parent” node and MTJ B as the “child” node; the child’s state is determined by the state of the parent through the weight of the connection between them. In our case, the weight is the controlled effect of dipole interaction which acts as a “wireless” connection. The weight is varied with stress generated electrically. Hardware implementation of Bayesian networks (or belief networks, in general) with MTJs has recently emerged as a topic of significant interest in view of the fact that these constructs are efficient computing machinery when computing in the presence of uncertainty (e.g. disease progression, stock market behavior, forecasting, computer vision, etc.)^31–34^. The present system offers an interesting energy-efficient way to implement a two-node Bayesian network, which can, in principle, be extended to multiple nodes by dipole-coupling multiple MTJs.

Bayesian networks would, of course, generally require both correlators and anti-correlators. To fashion a *correlator* out of two dipole-coupled MTJs (as opposed to the anti-correlator described here), one would simply place the two MTJs such that the line joining the centers of the elliptical soft layers is collinear with their major axes. In that case, dipole coupling would prefer “ferromagnetic ordering”, i.e. the magnetizations of the two soft layers will prefer to be mutually parallel. This is in contrast to the current situation where the line joining the centers of the elliptical soft layers is collinear with their minor axes. In the latter case, dipole coupling prefers “anti-ferromagnetic ordering”, i.e. the magnetizations of the two soft layers prefer to be mutually antiparallel. This is discussed in the Supplementary Material.

The effect of dipole coupling could also have been varied with a local magnetic field applied selectively on the second MTJ, or by driving a controlled amount of spin polarized current through the second MTJ, but these approaches are much more energy-inefficient than applying strain. It is also very difficult to generate a localized magnetic field that is confined to the second MTJ and does not infringe on the first. That is why strain is the preferred modality.

A natural question to ask at this point is why the resistance state of the MTJ A is also not set by strain since it is more energy efficient than other modalities. If we apply super-critical stress to the soft layer of the MTJ A, then that will align its magnetization along its minor axis (see Fig. 2) and upon stress release, the magnetization will relax to either orientation along the major axis with probabilities determined by dipole coupling with MTJ B and hence the bit state of MTJ B. This will not allow us to set *P(A)* to an arbitrary value (e.g. 70% probability of bit 0 and 30% of bit 1) *independent of B*. It is straightforward to see that applying critical stress will not ameliorate this problem. Second, the strain fields under MTJ A and MTJ B may interfere (since they are placed so close together) and this could be a spoiler. That is why it is preferable to set bit A with a voltage (generating STT) instead of using strain.

Note that the paradigm works for MTJ spacing spanning a fairly large range from 150 to 250 nm. For a 100-nm diameter MTJ, this will require the edge-to-edge separation to vary between 50 and 150 nm, which is very feasible. Hence, extreme lithographic precision is not required since there is a relatively large tolerance.

Finally, it is important to assess the importance of device variability and structural defects in the soft layers. Crystalline defects are irrelevant in our case since the soft layers are amorphous. Since the energy barrier is proportional to the nanomagnet volume, it is obvious that a few percent variability in the volume will induce the same percentage of change in the energy barrier. This will generally not impair device operation as long as the energy barrier is chosen to be much larger than the dipole coupling energy^26,27^. Thus reasonable (< 10%) variations in the MTJ’s soft layer thickness or lateral dimensions are not critical. As far as structural defects are considered, there will not be any significant effect from localized defects (e.g. a void in the material) either, but extended (delocalized) defects may be harmful^27^.

## Conclusion

p-bit streams with controlled amount of correlation have applications in various areas of probabilistic computing. While their traditional electronic implementation would require several components, here we have shown how they can be realized with just two dipole coupled magneto-tunneling junctions with magnetostrictive soft layers, fabricated on a piezoelectric film. This approach, which leverages coupled magneto-dynamics in two dipole-coupled MTJs, in one of which the switching probability under the influence of dipole coupling is modulated with strain, reduces device footprint, energy dissipation and cost. It also results in an “all-magnetic” platform for generating anti-correlations (using magnetic dipole coupling) while the modulation of the degree of anti-correlation and the read/write operations still require electrical components.

## Supplementary information

Supplementary Information.

## Data Availability

All data in the manuscript are available from the authors upon written request and subject to restrictions imposed by the authors’ institutions and funding agencies.
