# Supplementary material for: Electrically programmable probabilistic bit anti-correlator on a nanomagnetic platform
Source: Sci Rep. 2020 Jul 23;10:12361. doi: 10.1038/s41598-020-68996-y (PMC7378554; doi:10.1038/s41598-020-68996-y)
Supplement: Supplementary file 1 — Supplementary Information. [file 41598_2020_68996_MOESM1_ESM.pdf]

Supplementary Material: Electrically Programmable Probabilistic Bit Anti-Correlator on a Nanomagnetic Platform

Mason T. McCray, Md Ahsanul Abeed, and Supriyo Bandyopadhyay
Department of Electrical and Computer Engineering
Virginia Commonwealth University, Richmond, VA 23284, USA

Generation of controlled probability with spin-polarized current in MTJ A

Fig. S1 shows the soft layer of MTJ A schematically. We initialize the magnetization state of this layer with a very large spin polarized current which makes the magnetization point in the direction that represents bit 0 (i.e. $\theta = 0^0$ or $m_z = +1$). Next, varying magnitude of spin polarized current, with spin polarized along the direction representing bit 1, is injected perpendicular to the plane of the layer to make the magnetization point in the direction representing bit 1 (i.e. $\theta = 180^0$ or $m_z = -1$) with varying probability. The probability depends on the magnitude of the current and we calculate the probability as a function of the current's magnitude.

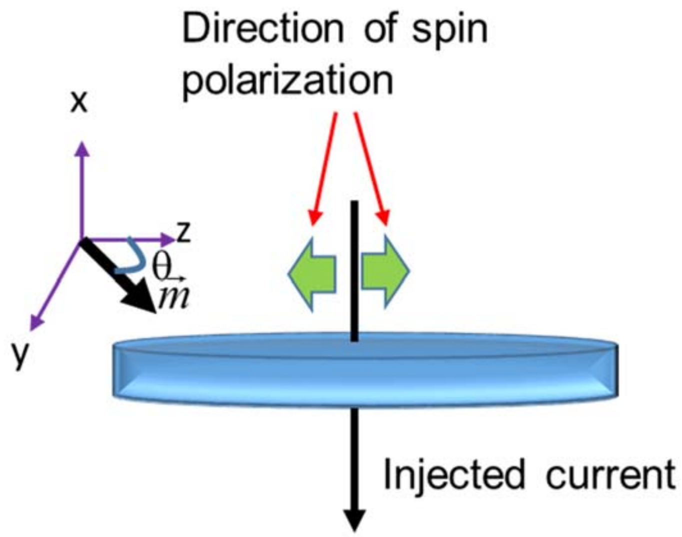

Fig. S1: Soft layer of MTJ A

The probability is calculated by simulating the magneto-dynamics in the soft layer using the stochastic Landau-Lifshitz-Gilbert equation which is Equation (1) in the text, except in this case, there is no stress $\left(\vec{H}_{\text{stress}} = 0\right)$ and we neglect dipole interaction $\left(\vec{H}_{\text{dipole}} = 0\right)$. Since the spin polarized current through the MTJ will be the dominant determinant of magneto-dynamics, we can ignore the dipole field.

Our initial conditions are: $m_x(0) = m_y(0) = 0;\ m_z(0) = +1$, i.e. the magnetization is initially pointing in the direction representing the bit 0. Current with spin polarized in the opposite direction along the major axis is injected and we calculate the probability that the magnetization flips to represent bit 1. This is the probability *P(1)*. We run the simulation following Equation (1) of the text until steady state is reached and the magnetization has settled to one of the two stable orientations along the major (easy) axis, i.e. $\theta \approx 0^0$ or 180^0. If $\theta \approx 180^0$, then we interpret the magnetization state to represent the bit 1; otherwise if $\theta \approx 0^0$, we interpret it as bit 0. We run 10,000 simulations and the probability of bit 1 is the fraction of simulations where the final state ends up at $\theta \approx 180^0$, i.e. as bit 1.

Fig. S2(a) plots the probability of MTJ A to be in the state representing bit 1, i.e. *P(1)*, as a function of the spin polarized current magnitude. Clearly, we can control the probability to be anything between 0% and 100% with the magnitude of the spin polarized current.

We have also examined a situation where the initial condition is $m_x(0) = m_z(0) = 0;\ m_y(0) = 1$, i.e. the magnetization of the soft layer of MTJ A is initially made to point along the minor (hard) axis. This can be ensured with an in-plane magnetic field in the direction of the minor axis that is initially turned on and then turned off. Fig. S2(b) plots the probability in this case as a function of the magnitude and *sign* of the spin polarized current. The sign is positive when the spin polarization is in the direction representing bit 1 and negative when it represents bit 0. In this case, we ran only 1000 simulations. Note that in this case, we need a much lower magnitude of the spin polarized current (an order of magnitude lower), which will reduce the energy dissipation. The disadvantage is that we will need to turn on and off an in-plane magnetic field each time we reset the probability generator. To produce the magnetic field will also require dissipating some energy.

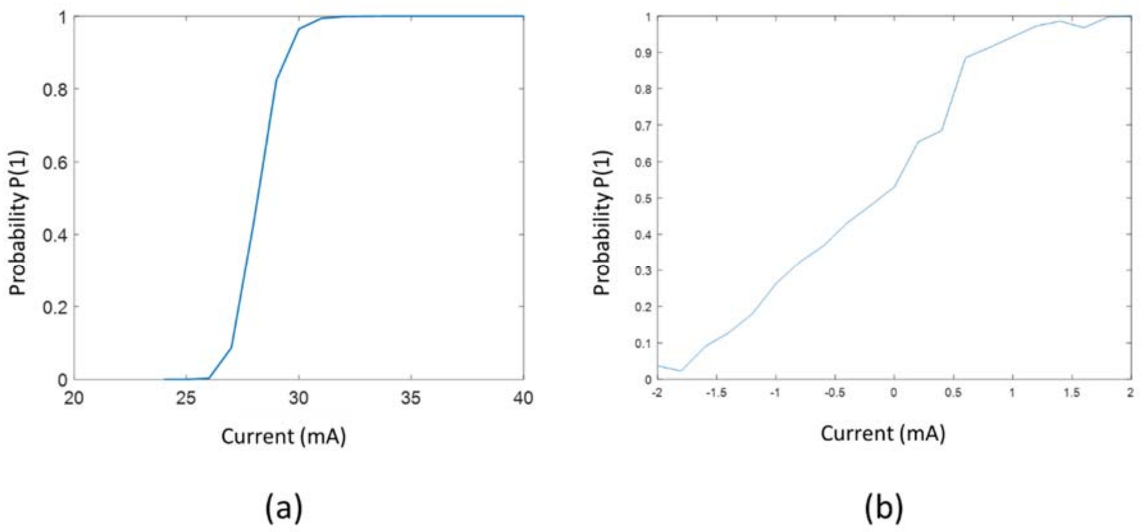

Fig. S2: Probability of bit 1 as a function of spin polarized current for two different initial conditions: (a) $m_x(0) = m_y(0) = 0;\ m_z(0) = +1$, and (b) $m_x(0) = m_z(0) = 0;\ m_y(0) = 1$.

Figs. S3(a) – S4(d) show the m_z trajectories as a function of time for four different magnitudes of the current in the case when the initial conditions are $m_x(0) = m_y(0) = 0;\ m_z(0) = +1$. Here, we have plotted all 10,000 trajectories. These plots show that the time scale for probability generation is no more than 5 ns.

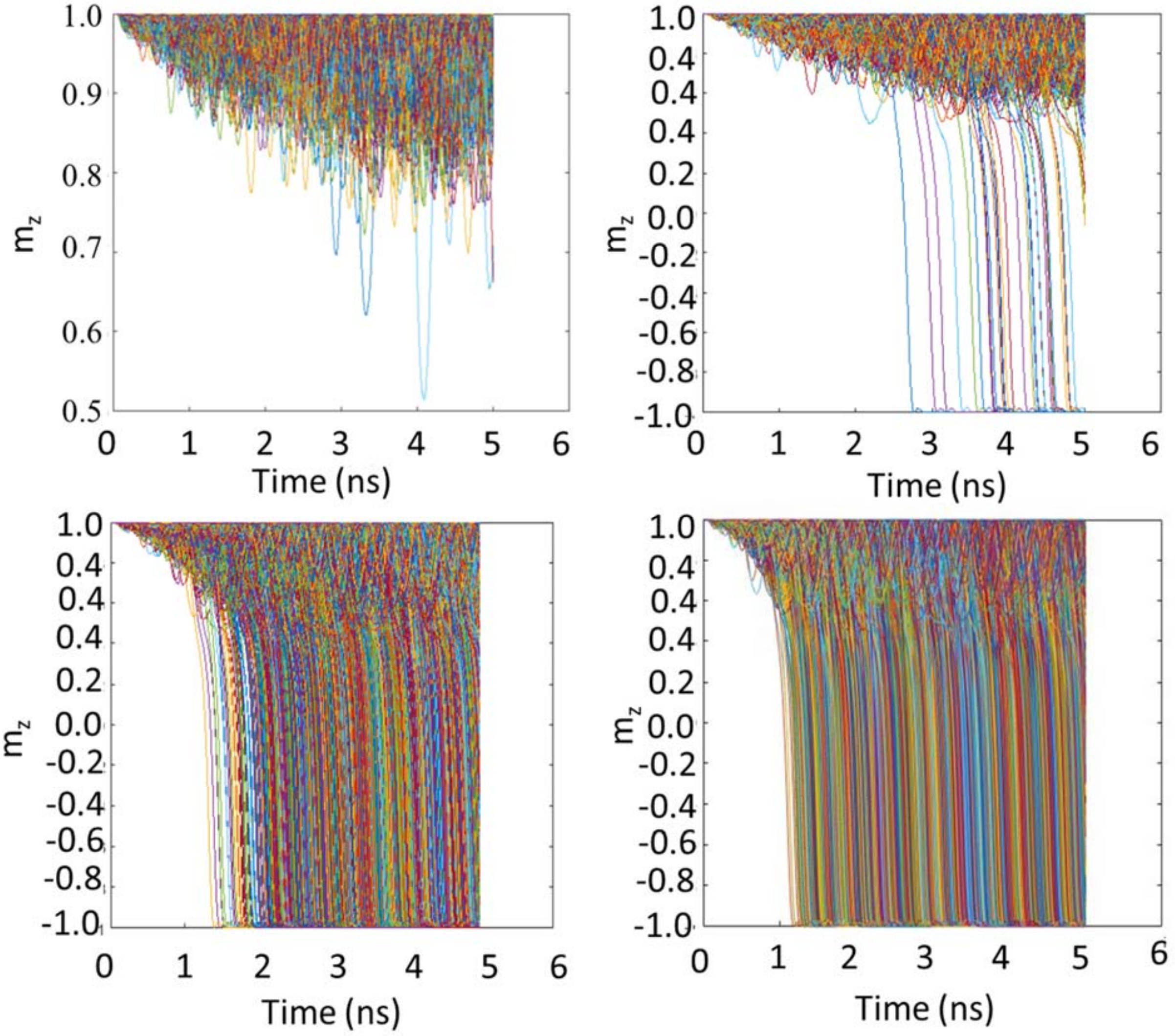

Fig. S3: Magnetization component along the major axis as a function of time for various magnitudes of spin polarized current: (a) 25 mA, (b) 26 mA, (c) 28 mA and (d) 30 mA.

Exchange coupled MTJ for high magnetostriction and high tunneling magneto-resistance ratio (TMR)

In our simulation, we considered an MTJ whose soft layer is made of Terfenol-D which provides high magnetostriction. This reduces the voltage needed to produce a given amount of strain in the piezoelectric and hence in the soft layer. Ultimately, that reduces the energy required to modulate the correlation.

However, the TMR of MTJs with Terfenol-D soft layer is not known. The highest TMRs are obtained in MTJs with CoFeB/MgO/CoFeB layers. One can engineer the best of both worlds by fabricating the structure shown below:

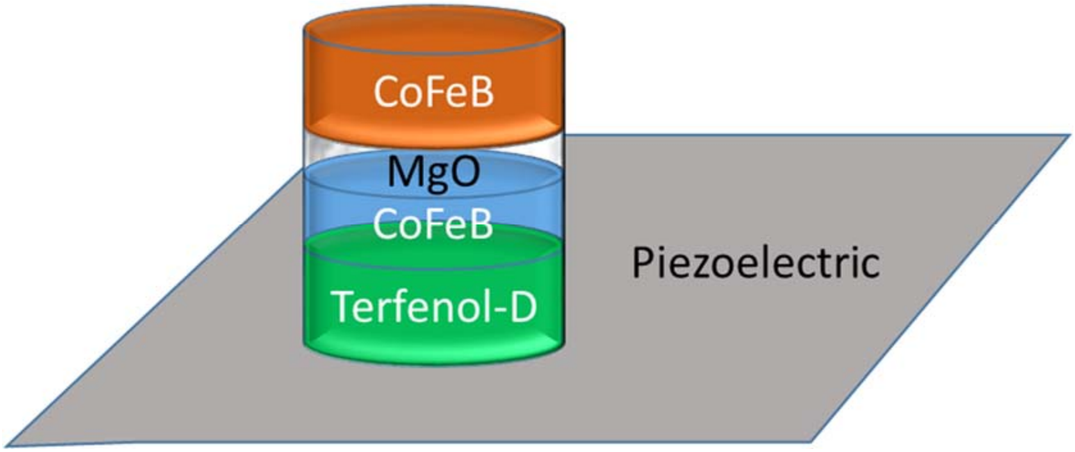

Fig. S4: An exchange coupled MTJ

In this structure, the bottom Terfenol-D and CoFeB layers are *exchange coupled* and hence their magnetization rotations are synchronized, i.e. when one rotates, the other does too. There could, however, be a potential lattice mismatch issue between Terfenol-D and CoFeB. An ultrathin texture break interlayer, e.g. Ta, which could maintain the exchange coupling between Terfenol-D and CoFeB, can be used to further optimize the performance of the composite soft layer (CoFeB/Ta(0.6-0.8nm)/Terfenol-D). This idea has been proposed and demonstrated (albeit with a different material system), and is used in today's STT-RAM product [1].

Correlator versus anti-correlator

If MTJ A and MTJ B are placed such that the line joining the centers of their elliptical soft layers is collinear with their minor axes (hard axes), as shown in Fig. S5(a), then dipole coupling prefers "anti-ferromagnetic ordering", i.e. the magnetizations of the two layers tend to be mutually antiparallel. This would lead to an *anti-correlator*. However, if MTJ A and MTJ B are placed such that the line joining the centers of their elliptical soft layers is collinear with their major axes (easy axes), as shown in Fig. S5(b), then dipole coupling prefers "ferromagnetic ordering", i.e. the magnetizations of the two layers tend to be mutually parallel. This would lead to a *correlator*. The system is not reconfigurable, i.e. the same pair cannot be made to act as either a correlator, or an anti-correlator, at will. However, this is not needed for most applications in probabilistic computing and belief networks.

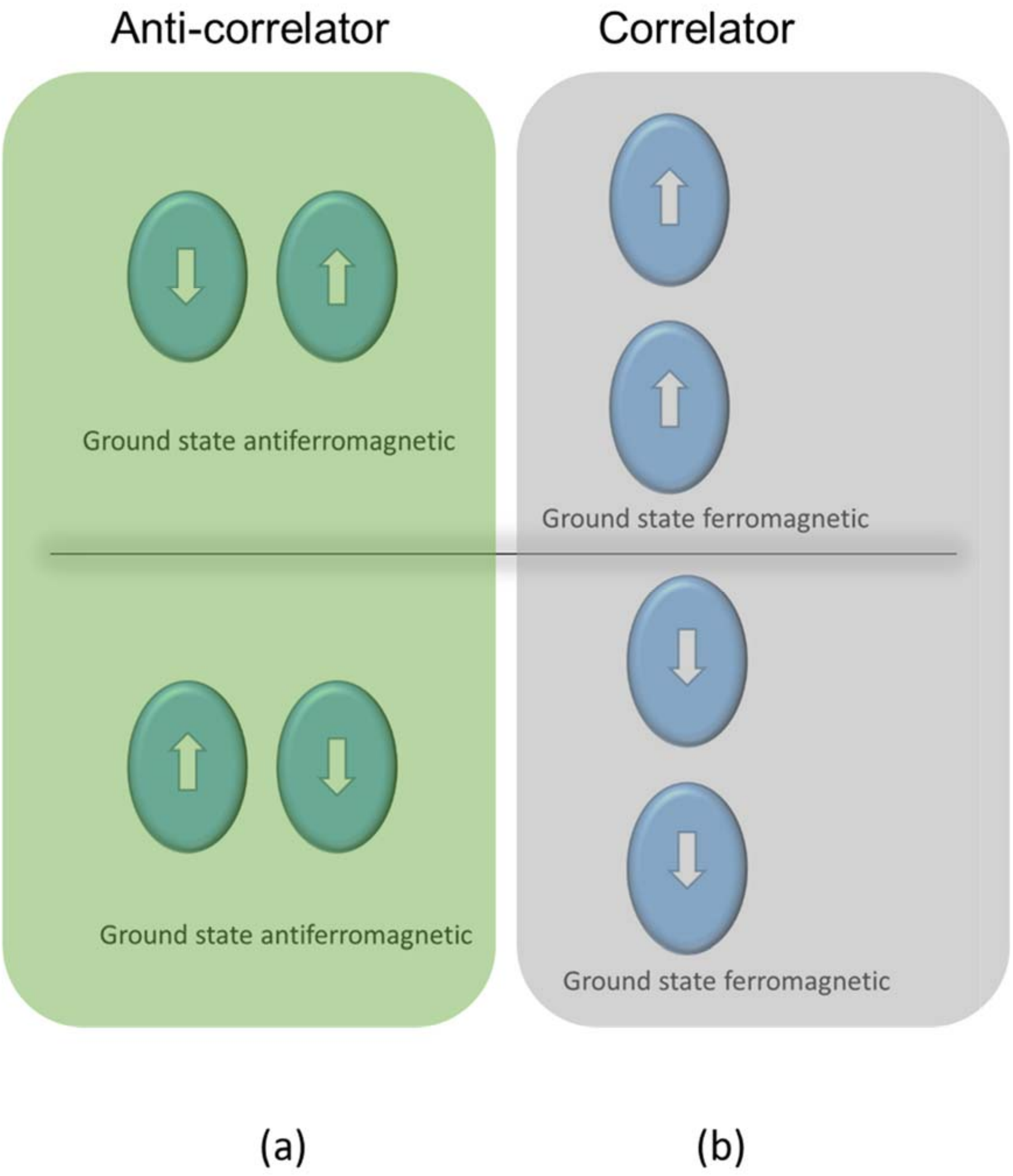

Fig. S5: The elliptical soft layers of MTJ A and MTJ B can be placed in two different ways with respect to each other. One configuration would produce an anti-correlator and the other a correlator.

Reference

1. Wang, J. P. & Rahman, M. T., US Patent 8,604,572.
